# Correction: Cardiorespiratory demands of firearms training instruction and 15m shuttle tests in British law enforcement

**DOI:** 10.1371/journal.pone.0321693

**Published:** 2025-04-01

**Authors:** Joseph Warwick, Sophie Cooper, Flaminia Ronca

The Data Availability statement for this article is incorrect. The correct statement is: Data cannot be shared publicly because of a confidentiality agreement with the large British police service. Data are available from the UCL Institutional Data Access/ Ethics Committee, contact via corresponding author for researchers who meet the criteria for access to confidential data or by emailing ethics@ucl.ac.uk and referencing project ID: 13985/004.
